# Nonpathogenic Cutibacterium acnes Confers Host Resistance against Staphylococcus aureus

**DOI:** 10.1128/Spectrum.00562-21

**Published:** 2021-10-27

**Authors:** Ayano Tsuru, Yumi Hamazaki, Shuta Tomida, Mohammad Shaokat Ali, Tomomi Komura, Yoshikazu Nishikawa, Eriko Kage-Nakadai

**Affiliations:** a Graduate School of Human Life Science, Osaka City University, Osaka, Japan; b Center for Comprehensive Genomic Medicine, Okayama University Hospital, Okayama, Japan; c Faculty of Human Sciences, Tezukayamagakuin University, Osaka, Japan; Lerner Research Institute

**Keywords:** *Caenorhabditis elegans*, *Cutibacterium acnes*, p38 MAPK, *Staphylococcus aureus*, *tir-1*

## Abstract

Cutibacterium acnes is a human skin-resident bacterium. Although C. acnes maintains skin health by inhibiting invasion from pathogens like Staphylococcus aureus, it also contributes to several diseases, including acne. Studies suggest that differences in genetic background may explain the diverse phenotypes of C. acnes strains. In this study, we investigated the effects of C. acnes strains on the Caenorhabditis elegans life span and observed that some strains shortened the life span, whereas other strains, such as strain HL110PA4, did not alter it. Next, we assessed the effects of C. acnes HL110PA4 on host resistance against S. aureus. The survival time of C. acnes HL110PA4-fed wild-type animals was significantly longer than that of Escherichia coli OP50 control bacterium-fed worms upon infection with S. aureus. Although the survival times of worms harboring mutations at the *daf-16*/FoxO and *skn-1*/Nrf2 loci were similar to those of wild-type worms after S. aureus infection, administration of C. acnes failed to improve survival times of *tir-1*/SARM1, *nsy-1*/mitogen-activated protein kinase kinase kinase (MAPKKK), *sek-1*/mitogen-activated protein kinase kinase (MAPKK), and *pmk-1*/p38 mitogen-activated protein kinase (MAPK) mutants. These results suggest that the TIR-1 and p38 MAPK pathways are involved in conferring host resistance against S. aureus in a C. acnes-mediated manner.

**IMPORTANCE** Cutibacterium acnes is one of the most common bacterial species residing on the human skin. Although the pathogenic properties of C. acnes, such as its association with acne vulgaris, have been widely described, its beneficial aspects have not been well characterized. Our study classifies C. acnes strains based on its pathogenic potential toward the model host C. elegans and reveals that the life span of C. elegans worms fed on C. acnes was consistent with the clinical association of C. acnes ribotypes with acne or nonacne. Furthermore, nonpathogenic C. acnes confers host resistance against the opportunistic pathogen Staphylococcus aureus. Our study provides insights into the impact of C. acnes on the host immune system and its potential roles in the ecosystem of skin microbiota.

## INTRODUCTION

The skin surface harbors a complex microbial ecosystem known as the skin microbiota (1). Cutibacterium acnes, a Gram-positive, aerotolerant anaerobic bacterium (2), is one of the most common and abundant bacterial species residing on the human skin (3, 4), particularly in sebaceous areas (1). It is associated with acne vulgaris, a chronic inflammatory disease of the pilosebaceous unit, affecting more than 85% of adolescents and young adults (5). However, C. acnes likely maintains skin health by inhibiting invasion of the skin tissues by common pathogens like Staphylococcus aureus (6).

At the strain level, C. acnes has been classified into three major genetic lineages, types I, II, and III (7, 8). These types are further subdivided into closely related clusters (IA-1, IA-2, IB, IC, II, and III) based on *recA* or into I-1a, I-1b, I-2, II, and III as determined by multilocus sequence typing (9–12). Moreover, some genetic lineages are associated with diseases, including acne (10–12). In an alternative approach, each C. acnes strain was assigned an “acne index” by calculating its prevalence in patients with acne using 16S rDNA metagenomic sequencing. Ribotype 4 (RT4), RT5, RT8, and RT10 were significantly enriched in patients with acne, whereas RT6 was found mostly in individuals without acne (13). The clinical association of C. acnes strains with disease or health has been corroborated experimentally *in vitro* and in a murine model, in which the application of acne-associated C. acnes strains (type IA2, RT4/5) led to the development of moderate-to-severe skin pathology and induced the production of proinflammatory cytokines compared with the application of health-associated type II (RT2/6) C. acnes strains (14).

Caenorhabditis elegans, a free-living nematode that feeds on bacteria, is an excellent model organism because it is easy to cultivate, has a short and reproducible life span, and tools for its genetic manipulation are available (15). C. elegans has emerged as a powerful genetic model for studying innate immunity, such as host response to infection (16), and has been extensively used as an alternative model host for investigating the effects of human intestinal microbiota. We have previously shown that aerobic lactobacillus- or anaerobic-bifidobacterium-fed C. elegans worms have increased resistance to Salmonella enterica (17) and Legionella pneumophila (18). Studies investigating the interactions between worms and their intestinal microbiota have revealed mechanisms by which bacteria augment immune responses and confer resistance to pathogens (19–22).

C. elegans has been used as a model for studying human skin microbiota. Aubin et al. used a C. elegans model to assess C. acnes virulence (23). Huang et al. identified pathogenic C. acnes strains and reported that the p38 mitogen-activated protein kinase (MAPK) pathway mediated host defense to C. acnes infection in C. elegans (24). Although the pathogenic properties of C. acnes strains have been described, their beneficial aspects have not been characterized. In this study, we classified C. acnes strains based on their pathogenicity toward C. elegans and examined the effect of a nonpathogenic C. acnes strain on host resistance to S. aureus in C. elegans.

## RESULTS

### Effects of various *C. acnes* strains on the life span of C. elegans.

**FIG 1:**
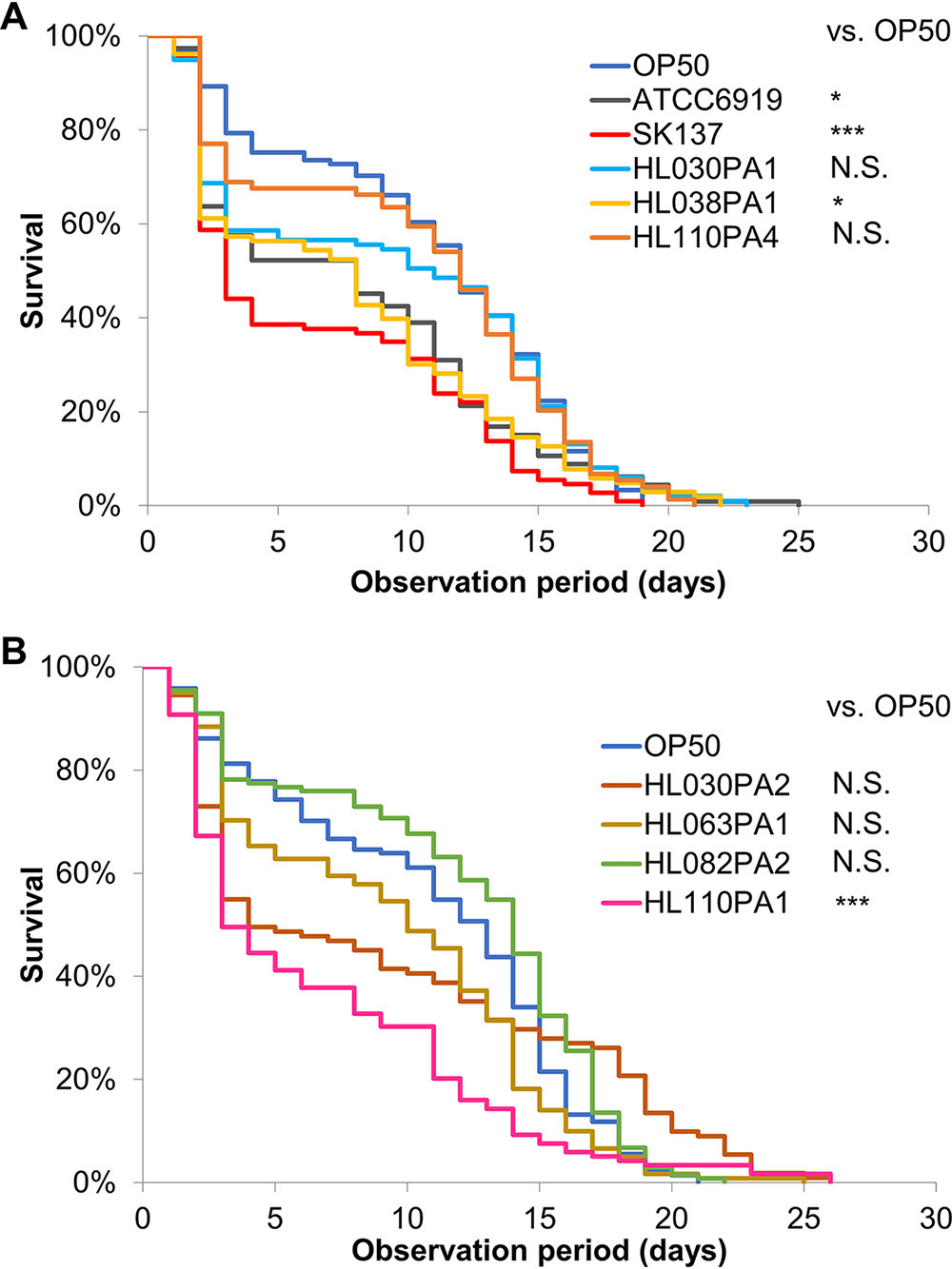
Diverse effects of Cutibacterium acnes strains on the life span of Caenorhabditis elegans. (A, B) Survival curves of worms fed Escherichia coli OP50 (control) or C. acnes strains. Worms were 3 days old on nominal day zero. (A) E. coli OP50-fed group (control), *n* = 121; ATCC 6919-fed group, *n* = 113; SK137-fed group, *n* = 109; HL030PA1-fed group, *n* = 99; HL038PA1-fed group, *n* = 103; and HL110PA4-fed group, *n* = 74. (B) E. coli OP50-fed group (control), *n* = 144; HL030PA2-fed group, *n* = 111; HL063PA1-fed group, *n* = 121; HL082PA2-fed group, *n* = 133; and HL110PA1-fed group, *n* = 119. Survival curves of worms fed C. acnes strains were compared with those of worms fed E. coli OP50. Differences in survival were tested for significance using the log-rank test. ***, *P < *0.001; *, *P < *0.05; N.S., not significant. Detailed life span data and statistics are provided in Table S6.

To examine the effects of C. acnes strains with diverse genetic backgrounds on the life span of C. elegans, we tested a series of C. acnes strains, namely, ATCC 6919, HL063PA1, SK137, HL038PA1, HL110PA1, HL030PA2, HL030PA1, HL082PA2, and HL110PA4 (Table 1). Notably, the life spans of ATCC 6919-, SK137-, HL038PA1-, and HL110PA1-fed worms were significantly shorter than those of Escherichia coli OP50-fed worms (*P = *0.01894, *P = *4.661 × 10^−6^, *P = *0.01089, and *P = *3.491 × 10^−5^, respectively) (Fig. 1A and B and Table 1; Table S6 in the supplemental material). In contrast, the life spans of animals fed other C. acnes strains were not significantly different from those of control animals (Fig. 1A and B and Table 1; Table S6). The former strains, those that shortened the worm’s life span, were defined as “pathogenic,” and the latter ones were defined as “nonpathogenic” strains.

**TABLE 1:** Cutibacterium acnes strains used in this study

Strain	Alternative name	Clade (13)	Ribotype (enriched in individuals with/without acne) (13)	Origin/description/source (13, 27)^*a*^	Shortened life span (this study)
ATCC 6919	NBRC107605	IA	RT1 (with/without acne)	Facial acne/IA type strain/NBRC	Yes
SK137	HM-122	IA-2	RT1 (with/without acne)	Normal skin of the right arm of a 57-yr-old man/pathogenicity island/BEI Resources	Yes
HL030PA1	HM‐504	IB-3	RT1 (with/without acne)	Nasal skin/HMP/BEI Resources	No
HL030PA2	HM‐505	IB-2	RT3 (with/without acne)	Nasal skin/HMP/BEI Resources	No
HL038PA1	HM‐512	IA-2	RT4 (with acne)	Nasal skin/HMP/BEI Resources	Yes
HL063PA1	HM‐528	IA-1	RT1 (with/without acne)	Nasal skin/HMP/BEI Resources	No
HL082PA2	HM‐536	II	RT2 (with/without acne)	Nasal skin/HMP/BEI Resources	No
HL110PA1	HM‐552	IB-1	RT8 (with acne)	Nasal skin/HMP/BEI Resources	Yes
HL110PA4	HM‐555	II	RT6 (without acne)	Nasal skin/HMP/BEI Resources	No

a HMP, Human Microbiome Project.

### Genes related to the GO term “defense response to Gram-positive bacteria” were upregulated by nonpathogenic *C. acnes*.

**FIG 2:**
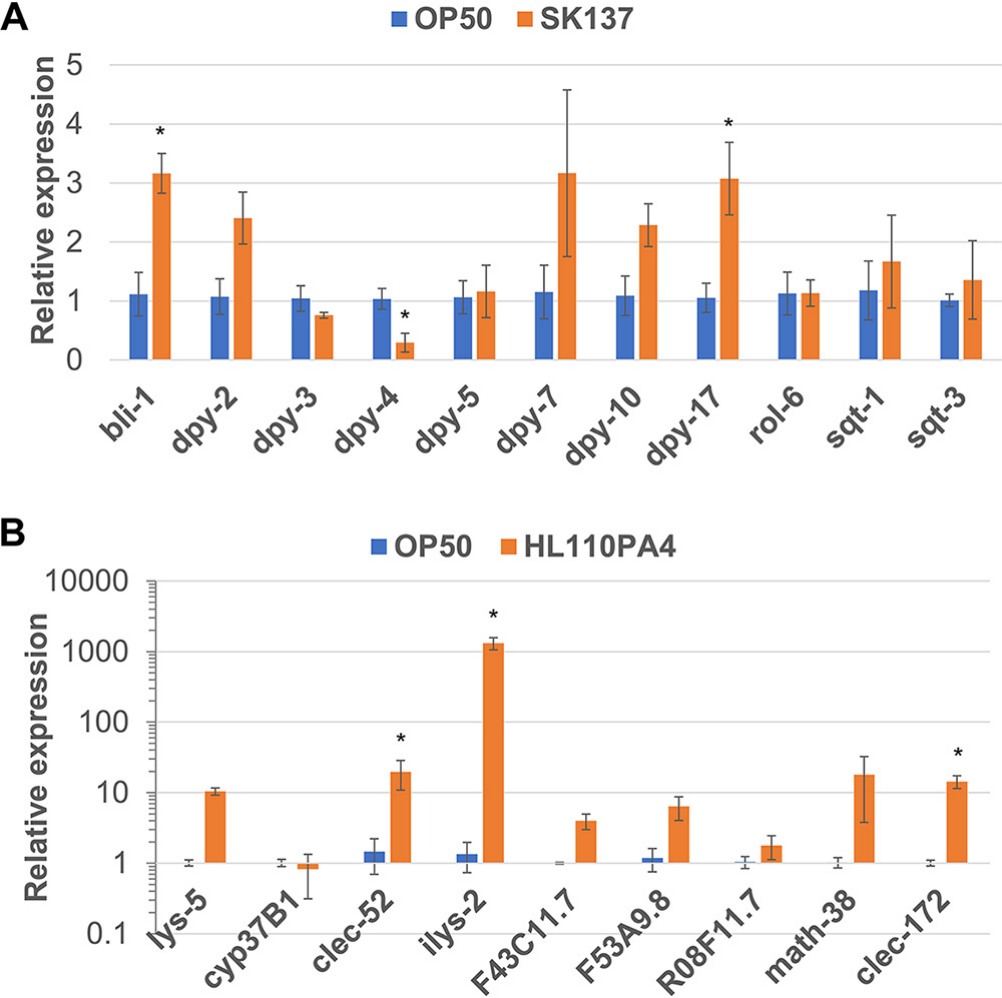
Expression of genes related to top-ranked GO terms quantified using real-time PCR. (A) Expression of genes related to “cuticle development involved in collagen and cuticulin-based cuticle molting cycle” that were enriched in the SK137-fed group. Relative mRNA levels (normalized to *act-1* and *tba-1*) in the animals fed with SK137 are presented in comparison with the control group (E. coli OP50). (B) Expression of genes related to “defense response to Gram-positive bacterium” that were enriched in the HA110PA4-fed group. Relative mRNA levels (normalized to *cyc-1*, *act-1*, and *tba-1*) in the animals fed with HA110PA4 are presented in comparison with the control group (E. coli OP50). (A, B) Error bars represent the standard error (SEs) of the results of three independent experiments. Statistical analyses were performed using a two-tailed *t* test. *, *P < *0.05 versus control.

C. acnes infection has been shown to induce the expression of antimicrobial molecules like C-type lectins and lysozymes in C. elegans (24). To characterize the genes activated by pathogenic or nonpathogenic C. acnes strains in C. elegans, RNA sequencing was performed. SK137 and HL110PA4 were used as representative pathogenic and nonpathogenic strains, respectively, and E. coli OP50 was used as a control. A total of 493 genes showed increased expression in the SK137-fed group relative to the gene expression in the E. coli OP50 (control)-fed group, with a log_2_ fold change (FC) of ≥2 and *P* value of <0.05 (Table S1). Significantly enriched Gene Ontology (GO) terms in the SK137-upregulated genes are listed in Table S3. Among the enriched GO terms for biological processes (BPs) (Table 2), the top-ranked one was “cuticle development involved in collagen and cuticulin-based cuticle molting cycle” (*P* = 8.02 × 10^−13^) (Table S3). The levels of mRNA expression for *bli-1*, *dpy-2*, *dpy-3*, *dpy-4*, *dpy-5*, *dpy-7*, *dpy-10*, *dpy-13*, *dpy-17*, *rol-6*, *sqt-1*, and *sqt-3*, which are related to the GO term, were evaluated using real-time PCR. *dpy-13* was excluded because it was not detected by real-time PCR. Of the 11 genes, the expression levels of *bli-1* and *dpy-17* mRNA were significantly higher in the SK137-fed group than in the control group (*P* = 0.0150 and *P* = 0.0376, respectively) (Fig. 2A). In contrast, the expression levels of 248 genes increased in the HL110PA4-fed group (Table S2), with the top enriched GO term being “defense response to Gram-positive bacterium” (*P* = 3.47 × 10^−11^) (Table 3; Table S4). The levels of mRNA expression of the 10 genes, namely, *lys-5*, *spp-2*, *cyp-37B1*, *clec-52*, *ilys-2*, *F43C11.7*, *F53A9.8*, *R08F11.7*, *math-38*, and *clec-172*, were quantified using real-time PCR. Of the nine genes (*spp-2* was excluded because it was not detected in real-time PCR), the expression levels of *lys-5* and *clec-172* were significantly higher in the HL110PA4-fed group than in the E. coli OP50 (control)-fed group (*P* = 0.0174 and *P* = 0.0449, respectively) (Fig. 2B).

**TABLE 2:** Gene Ontology terms for biological processes that were significantly enriched in genes upregulated by *C. acnes* strain HM-122

GO_ID	GO_term/description	*P* value	*q* value
0042338	Cuticle development involved in collagen and cuticulin-based cuticle molting cycle	8.02e−13	5.99e−10
0018996	Molting cycle, collagen and cuticulin-based cuticle	3.85e−10	1.12e−07
0042303	Molting cycle	4.51e−10	1.12e−07
0098542	Defense response to other organism	2.38e−08	3.15e−06
0009607	Response to biotic stimulus	2.95e−08	3.15e−06
0043207	Response to external biotic stimulus	2.95e−08	3.15e−06
0051707	Response to other organism	2.95e−08	3.15e−06
0030855	Epithelial cell differentiation	3.96e−06	0.000227
0006952	Defense response	4.87e−06	0.00026
0010466	Negative regulation of peptidase activity	6.36e−05	0.002639
0045861	Negative regulation of proteolysis	6.36e−05	0.002639
0007160	Cell-matrix adhesion	9.54e−05	0.003567
0031589	Cell-substrate adhesion	9.54e−05	0.003567
0043086	Negative regulation of catalytic activity	0.00014	0.004754
0052547	Regulation of peptidase activity	0.00026	0.007208
0051172	Negative regulation of nitrogen compound metabolic process	0.000308	0.007944
0044092	Negative regulation of molecular function	0.000309	0.007944
0120036	Plasma membrane-bounded cell projection organization	0.000319	0.007944
0031324	Negative regulation of cellular metabolic process	0.000427	0.009756
0030030	Cell projection organization	0.000436	0.009756
1905515	Nonmotile cilium assembly	0.000561	0.011975
0034608	Vulval location	0.001173	0.023065
0060429	Epithelium development	0.001598	0.030628
0000122	Negative regulation of transcription from RNA polymerase II promoter	0.001867	0.034026
0016042	Lipid-catabolic process	0.002169	0.035247

**TABLE 3:** Gene Ontology terms for biological processes that were significantly enriched in genes upregulated by *C. acnes* strain HM-555

GO_ID	GO_term/description	*P* value	*q* value
0050830	Defense response to Gram-positive bacterium	3.47e−11	1.22e−08
0006952	Defense response	5.40e−09	3.21e−07
0009607	Response to biotic stimulus	5.47e−09	3.21e−07
0040002	Collagen and cuticulin-based cuticle development	1.07e−05	0.000418
0016042	Lipid-catabolic process	9.38e−05	0.002756

### Nonpathogenic *C. acnes* HL110PA4 confers resistance against S. aureus.

**FIG 3:**
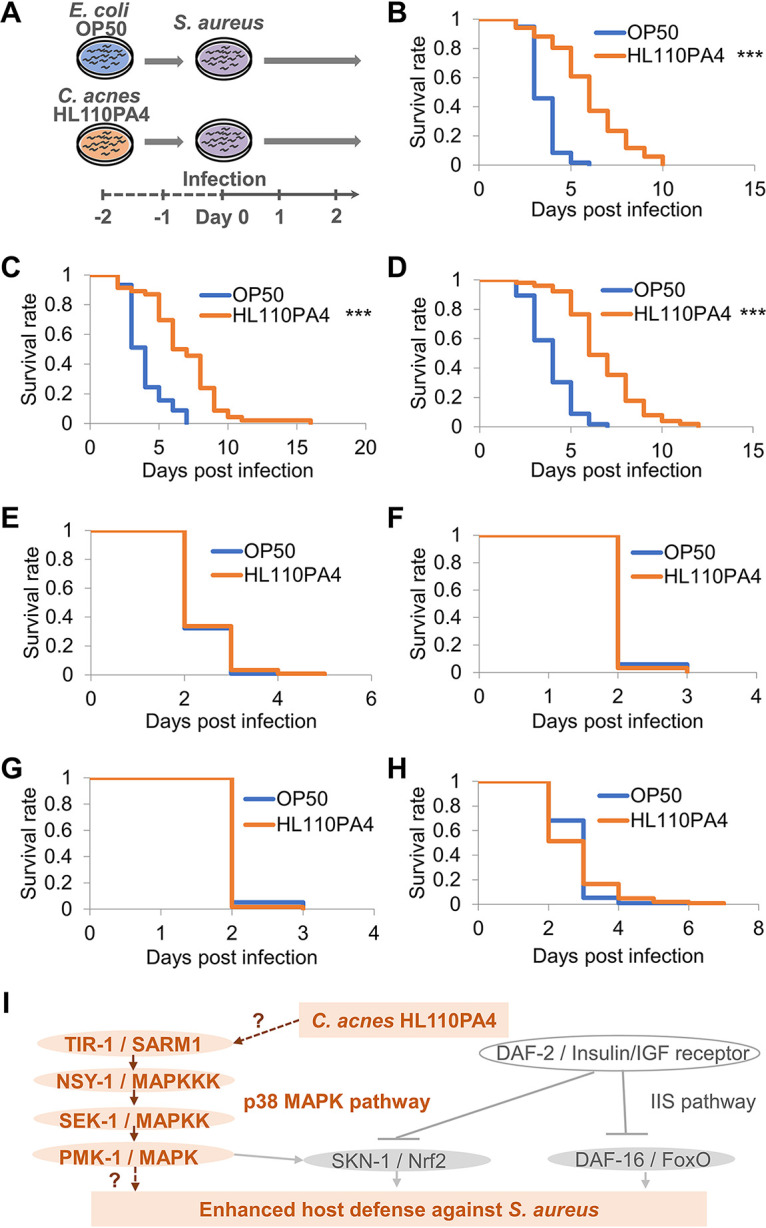
Nonpathogenic Cutibacterium acnes*-*mediated enhancement of worm survival time upon Staphylococcus aureus infection depends on *tir-1* and p38 MAPK pathway. (A) Diagram illustrating the worm survival assay upon S. aureus infection. Worms were fed E. coli OP50 (control) or C. acnes HL110PA4 for 2 days and subsequently infected with S. aureus (day zero). (B to H) Survival of wild-type (WT) and mutant worms infected with S. aureus. Survival curves of the wild-type N2, E. coli OP50-fed group (control), *n* = 40, and HL110PA4-fed group, *n* = 47 (B), *daf-16*(*mu86*) mutant, E. coli OP50-fed group (control), *n* = 40, and HL110PA4-fed group, *n* = 42 (C), *skn-1*(*ok2315*), E. coli OP50-fed group (control), *n* = 55, and HL110PA4-fed group, *n* = 49 (D), *tir-1*(*qd4*), E. coli OP50-fed group (control), *n* = 45, and HL110PA4-fed group, *n* = 39 (E), *nsy-1*(*ag3*), E. coli OP50-fed group (control), *n* = 23, and HL110PA4-fed group, *n* = 30 (F), *sek-1*(*km4*) E. coli OP50-fed group, (control), *n* = 58, and HL110PA4-fed group, *n* = 56 (G), and *pmk-1*(*km25*), E. coli OP50-fed group (control), *n* = 113, and HL110PA4-fed group, *n* = 103 (H). These survival curves were compared with those of worms fed E. coli OP50. Differences in survival were tested for significance using the log-rank test. ***, *P < *0.001. Detailed life span data and statistics are provided in Table S6. (I) Working model for the enhanced host defense mediated by C. acnes HL110PA4. This study demonstrated that C. acnes-mediated host resistance against S. aureus requires the TIR-1/SARM1 and p38 MAPK cascade but not SKN-1/Nrf2 or DAF-16/FoxO. Inductive pathways are shown as arrows, and suppressive pathways are shown as arrows with T-shaped heads.

Because nonpathogenic C. acnes HL110PA4 induced the expression of genes involved in defense against Gram-positive bacteria, we examined the effect of C. acnes HL110PA4 on host resistance to S. aureus, which is a Gram-positive, opportunistic pathogen and a human skin-resident microbe. Notably, worms fed C. acnes HL110PA4 exhibited prolonged survival compared with the survival of controls upon S. aureus infection (Fig. 3A and B; Table S6).

### Nonpathogenic *C. acnes* HL110PA4 extends the life span of worms upon S. aureus infection in a TIR-1 and p38 MAPK pathway-dependent manner.

The insulin/insulinlike growth factor 1 (IGF-1) signaling (IIS) and p38 MAPK pathways play central roles in innate immunity in C. elegans (16, 25). To investigate the involvement of the IIS and p38 MAPK pathways, the survival of loss-of-function mutants with mutations of *daf-16*/FOXO, *skn-1*/Nrf2, *tir-1*/SARM1, *nsy-1*/mitogen-activated protein kinase kinase kinase (MAPKKK), *sek-1*/mitogen-activated protein kinase kinase (MAPKK), and *pmk-1*/p38 MAPK was evaluated. Upon infection with S. aureus, the survival times of the *daf-16* and *skn-1* mutants fed nonpathogenic C. acnes strains were significantly longer than those of mutants in the E. coli OP50-fed control group (Fig. 3C and D), which were similar to the survival seen for wild-type animals (Fig. 3B). In contrast, feeding nonpathogenic C. acnes failed to improve survival times in S. aureus-infected *tir-1*, *nsy-1*, *sek-1*, or *pmk-1* mutants (Fig. 3E to H). The above-described results suggested that the extension of the survival times of S. aureus-infected worms depended on *tir-1*, *nsy-1*, *sek-1*, and *pmk-1* but not on *daf-16* and *skn-1* function (Fig. 3I).

### *C. acnes* does not directly inhibit the growth of S. aureus.

**FIG 4:**
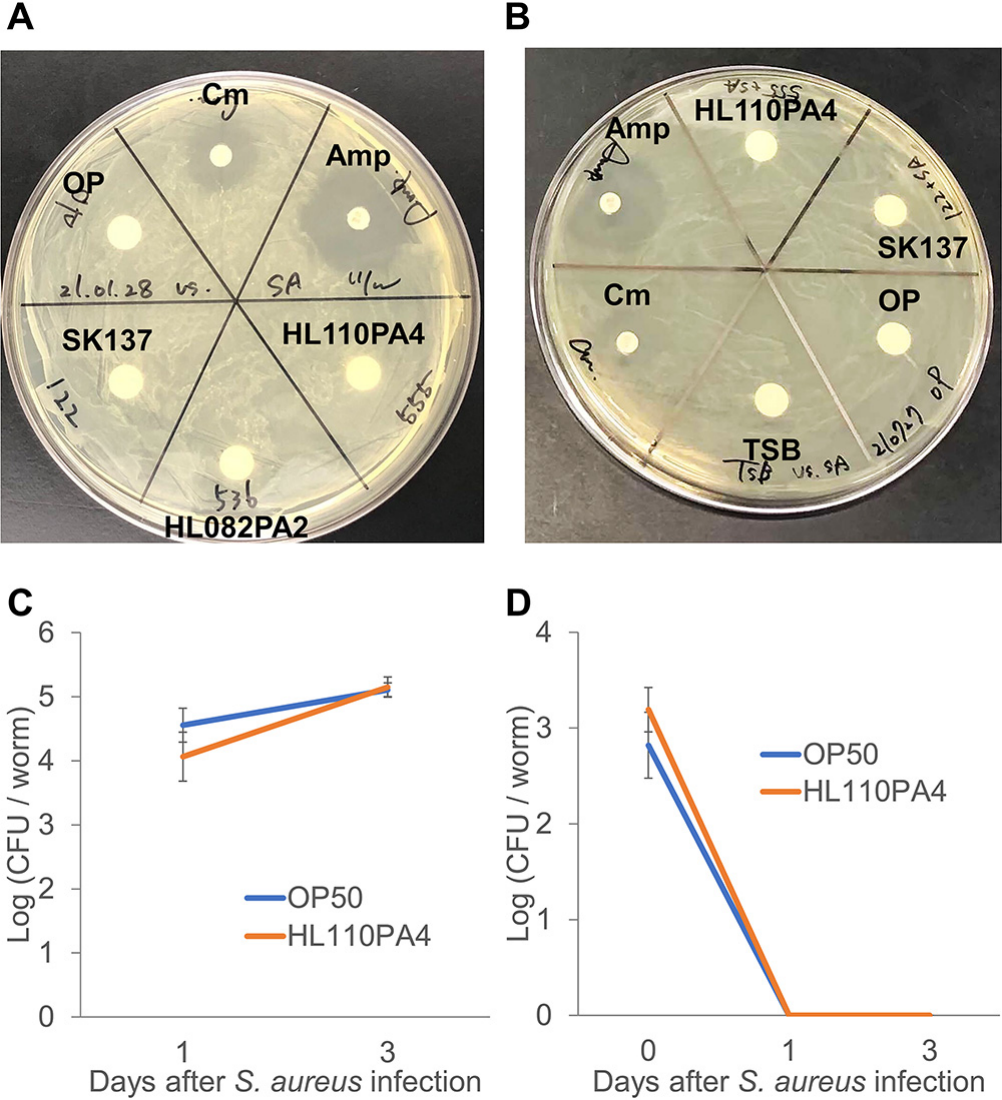
Disk diffusion assay and bacterial-cell recovery assay. (A) Growth inhibition of Staphylococcus aureus by cell-free supernatants of Cutibacterium acnes SK137 (HM-122), HL110PA4 (HM-555), or HL082PA2 (HM-536), Escherichia coli OP50 (OP), or the antibiotics (positive controls) chloramphenicol (Cm) and ampicillin (Amp) in the disk diffusion assay. (B) Growth inhibition of S. aureus by cell-free supernatant of mixed culture of C. acnes SK137 (HM-122), C. acnes HL110PA4 (HM-555), or E. coli OP50 with S. aureus. The antibiotics, chloramphenicol and ampicillin, were used as positive controls. Tryptone soy broth (TSB) was used as a negative control. (C) CFU of S. aureus recovered from worms infected with S. aureus in E. coli OP50 (control)- or HL110PA4-fed groups, *n* = 5. (D) CFU/worm of recovered E. coli OP50 or HL110PA4 in E. coli OP50 (control)- or HL110PA4-fed groups, respectively, upon infection with S. aureus, *n* = 5. Error bars indicate SE.

A disk diffusion assay was performed to determine whether C. acnes secretes antimicrobial molecules that inhibit S. aureus, such as bacteriocins and secondary metabolites. Cell-free supernatant of nonpathogenic C. acnes did not inhibit S. aureus (Fig. 4A) growth. As several bacteria are known to produce antimicrobial molecules only in the presence of another competing microbe, we tested the effect of the cell-free supernatant prepared from a mixed culture of C. acnes and S. aureus (Fig. 4B). However, no inhibitory effect was observed. Although several Gram-negative bacteria compete via contact-dependent systems called type VI secretion systems (T6SS), we confirmed that the T6SS sequence does not exist on the genome of the HL110PA4 C. acnes (GCA_000144895.1) (26). In addition, we observed no difference in the numbers of S. aureus cells recovered between E. coli OP50- and C. acnes-fed worms on each test date (Fig. 4C). Moreover, neither E. coli nor C. acnes was recovered from S. aureus-infected worms on days 1 and 3 after the infection (day zero) (Fig. 4D).

## DISCUSSION

Our findings revealed various effects of C. acnes strains of different ribotypes (RTs) on worm life spans. ATCC 6919 (RT1), SK137 (RT1), HL038PA1 (RT4), and HL110PA1 (RT8) shortened the life span of worms, whereas the other strains did not alter it. Based on the metagenomic analysis of the skin microbiome associated with acne (13), RT1 was found in individuals both with and without acne, with no significant differences, whereas RT4 and RT8 were enriched in patients with acne. Indeed, the worm life span was decreased by HL038PA1 and HL110PA1, which belong to the acne patient-associated RTs, RT4 and RT8, respectively. The decreased life spans observed in ATCC 6919- or SK137-fed worms were in agreement with the results of previous studies; ATCC 6919 has been reported to shorten the life span of C. elegans (24), and SK137 has been shown to possess pathogenicity islands in its genome (27). In contrast, the RT1 strains HL063PA1 and HL030PA1 did not shorten the life span, nor did HL082PA2 (RT2) or HL030PA2 (RT3). The ribotypes of the latter were also found in individuals with and without acne (13). HL110PA4, which belongs to RT6, which was found mostly in individuals without acne (13), also did not affect life span. Overall, the life span of C. elegans was consistent with the clinical association of C. acnes RTs with acne or nonacne.

As shown by the results in Fig. 1, C. acnes-fed worms tended to exhibit “early death,” wherein animals died earlier than observation day 5. The survival curve of the HL030PA2-fed group intersected with that of the control, suggesting a long survival time for the remaining animals. Multiple causes of death have been implicated according to previous studies; specifically, two classes of dead nematodes are observed, those succumbing to early death with a swollen pharynx (termed “P deaths”) and those succumbing to late death with an atrophied pharynx (termed “p deaths”) (28). As pharyngeal swelling is caused by bacterial infections, wherein bacteria invade the pharyngeal tissues, C. acnes may be able to invade the pharynx more effectively than E. coli and cause early deaths (P deaths). In addition, late deaths might be suppressed in HL030PA2-fed animals. Further characterization of the survival dynamics among the worm groups fed different C. acnes strains is warranted to address the variation in C. acnes-associated diseases in humans.

One potential limitation of the use of C. elegans as a model host for anaerobic microbes is the requirement for an aerobic environment. Although C. acnes is tolerant of aerobic conditions, they may impact the viability and/or life cycle of C. acnes and, in turn, affect its ability to influence the life span of worms. It should also be mentioned that strict anaerobes like Bifidobacterium infantis and Clostridium butyricum have been assessed in the aerobic C. elegans model and have been shown to extend the life span of worms (29, 30). In these studies, feeding bacterial-cell components or UV-inactivated bacteria also resulted in alterations in worm longevity (29, 30). Further investigations are required to address the utility of using C. elegans as a model organism to study the biological mechanisms prevailing in C. acnes and other anaerobes.

Based on the transcriptome analysis, the expression of genes related to the GO term “defense response to Gram-positive bacterium” increased in the presence of the nonpathogenic C. acnes strain HL110PA4. In contrast, the pathogenic strain SK137 induced the expression of genes related to the cuticle. SK137 may interact with the cuticle to exert its pathogenicity, although further investigations are warranted in this area. Importantly, Huang et al. (24) have determined that C. acnes induces genes encoding C-type lectins like *clec-52* and lysozymes like *lys-5*, as is the case with HL110PA4, but the other series of induced C-type lectins and lysozymes were different from those observed in HL110PA4. Although we chose two representative strains, HL110PA4 and SK137, for differential expression (DE) analysis and GO enrichment analysis in the present study, we propose the use of diverse C. acnes strains for cluster analysis, as it will facilitate the identification of markers that can discriminate between pathogenic and nonpathogenic strains.

Nonpathogenic C. acnes HL110PA4 enhanced the survival of worms infected with Gram-positive S. aureus, consistent with the results of transcriptome analysis. However, it is still unknown whether this effect on the survival of worms infected with S. aureus is specific to nonpathogenic strains. The IIS and p38 MAPK pathways play central roles in C. elegans infection (16). IIS is initiated by the binding of insulinlike peptides to the receptor DAF-2. This leads to the activation of downstream IIS kinases, which phosphorylate and inhibit the FOXO transcription factor DAF-16 in C. elegans (31–33). The p38 MAPK pathway, involving the MAPKKK/NSY-1, MAPKK/SEK-1, and MAPK/PMK-1 cascade, activates SKN-1 (an ortholog of mammalian Nrf2) in several contexts, such as pathogen infection in C. elegans (34–36); moreover, SKN-1 has been shown to be directly suppressed by DAF-2 (37). TIR-1, a TIR domain-containing adaptor protein, has been suggested to be an upstream component of the p38 MAPK pathway upon infection (38). Mutant analyses revealed that the extension of the survival times of S. aureus-infected worms required *tir-1*, *nsy-1*, *sek-1*, and *pmk-1* but not *daf-16* and *skn-1* function. Moreover, *nsy-1*, *sek-1*, and *pmk-1* are likely to be involved in the defense response against S. aureus. Huang et al. reported that the p38 MAPK cascade (NSY-1–SEK-1–PMK-1) mediates host defense against C. acnes infection in C. elegans (24). However, *tir-1* is not required for host defense mechanisms (24). The upstream and downstream elements of the TIR-1- and PMK-1-associated pathways remain unidentified, as well as their role in the worm’s C. acnes-mediated resistance to S. aureus. In mammals, Toll-like receptors (TLRs) recognize pathogens and initiate an innate immune response by recruiting intracellular adaptor proteins via heterotypic Toll/interleukin­1 receptor (TIR) domain interactions (39, 40). In C. elegans, however, *tol-1*, which encodes a sole orthologue for TLRs, exhibits limited effects on pathogen susceptibility and is unlikely to associate with *tir-1* (38, 41, 42). A candidate downstream factor of PMK-1 could be ATF-7, which functions as a transcriptional regulator of PMK-1-mediated innate immunity. ATF-7 has only been shown to be involved in infections of the Gram-negative bacterium Pseudomonas (43). In contrast, SKN-1 plays roles in infections of both Gram-positive and Gram-negative bacteria (36).

Grice and Segre proposed that C. acnes could inhibit the invasion of skin pathogens like S. aureus (6). In the present study, cell-free supernatant of C. acnes did not inhibit S. aureus. In addition, we observed no difference in the numbers of bacterial CFU recovered after infection with S. aureus between E. coli OP50- and C. acnes-fed worms on each test date. Furthermore, we observed that C. acnes was not recovered from worms after S. aureus infection, suggesting that direct inhibition of S. aureus by C. acnes is not the central mechanism in enhancing host resistance. Worms fed C. acnes appeared tolerant; that is, they were resistant to S. aureus without reducing the number of infecting bacterial cells.

In conclusion, nonpathogenic C. acnes conferred resistance to S. aureus through the TIR-1 and p38 MAPK pathways. However, our results were obtained from the invertebrate model host C. elegans. Hence, studies on the beneficial attributes of C. acnes and the mechanisms governing host defense in mammals and humans are warranted. Nevertheless, our study sheds light on the impact of C. acnes on the host immune system and the potential role of this bacterium in the ecosystem of skin microbiota.

## MATERIALS AND METHODS

### Bacterial strains and culture conditions.

Escherichia coli strain OP50, obtained from the Caenorhabditis Genetics Center, was used as the standard feed for nematode cultivation. C. acnes strains SK137 (HM‐122), HL038PA1 (HM‐512), HL030PA1 (HM‐504), HL110PA4 (HM‐555), HL082PA2 (HM‐536), HL110PA1 (HM‐552), HL030PA2 (HM‐505), and HL063PA1 (HM‐528), which were originally isolated from human nasal skin in the National Institutes of Health (NIH) Human Microbiome Project (HMP) (13) (except for SK137), were acquired from the Biodefense and Emerging Infections Research Resources Repository (BEI Resources, Manassas, VA, USA) (Table 1). C. acnes strain ATCC 6919 (NBRC107605) and S. aureus strain ATCC 6538 (NBRC13276) were obtained from the National Institute of Technology and Evaluation (Tokyo, Japan). E. coli and S. aureus were cultured in tryptone soy broth (TSB) and on tryptone soy agar (TSA) (Nissui Pharmaceutical, Tokyo, Japan) at 37°C. C. acnes strains were cultured in Gifu anaerobic medium (GAM) broth and on GAM agar (Nissui Pharmaceutical, Japan) under anaerobic conditions at 37°C. Cultured bacteria (100 mg [wet weight]) were scraped and weighed and then suspended in 0.5 ml M9 buffer (5 mmol/liter potassium phosphate, 1 mmol/liter CaCl_2_, and 1 mmol/liter MgSO_4_). Then, 50 μl bacterial suspension was spread on peptone-free modified nematode growth medium (mNGM) (1.7% [wt/vol] agar, 50 mmol/liter NaCl, 1 mmol/liter CaCl_2_, 5 μg/ml cholesterol, 25 mmol/liter KH_2_PO_4_, and 1 mmol/liter MgSO_4_) (10 mg bacteria per plate) in 5-cm diameter plates to feed the worms under aerobic conditions.

### C. elegans strains and culture conditions.

C. elegans Bristol strain N2 (wild-type) was obtained from the Caenorhabditis Genetics Center. The mutants used in this study included CF1038 *daf-16*(*mu86*), ZD101 *tir-1*(*qd4*), AU3 *nsy-1*(*ag3*), KU4 *sek-1*(*km4*), KU25 *pmk-1*(*km25*), and VC1772 *skn-1*(*ok2315*). Nematodes were maintained on NGM according to standard methods (Brenner [44]): briefly, eggs were suspended in M9 buffer for 1 day at 25°C to allow hatching and synchronization; the resulting suspension of synchronized L1-stage worms was centrifuged at 156 × *g* for 1 min. The supernatant was removed by aspiration, and the larvae were used for subsequent assays.

### Determination of C. elegans life span.

L1-stage worms were cultured on mNGM plates covered with E. coli OP50 at 25°C for 2 days until they reached the young-adult stage (referred to as 3-day-old animals). Three-day-old animals (35 animals per plate) were placed on 5-cm mNGM plates covered with 10 mg E. coli OP50 or C. acnes strains and then incubated at 25°C. Worms were transferred daily to fresh plates for the first 4 days and every second day thereafter. The numbers of live and dead animals were scored daily. An animal was considered dead when it failed to respond to gentle touch with a worm picker. Animals that crawled off the plate or died from internal hatching were considered lost and excluded from the analysis. Each assay was conducted in duplicate and repeated twice, unless otherwise stated. Worm survival was calculated using the Kaplan-Meier method, and survival differences were tested for significance using the log-rank test.

Mean life span (MLS) was estimated using the following formula (45):

$$\text{MLS}=\frac{1}{N}\underset{j}{\sum }\frac{x_{j}\;+\;x_{j+1}}{2}d_{j}$$

where *d_j_* is the number of worms that died in the age interval (*x_j_* to *x_j+1_*) and *N* is the total number of worms. The standard error (SE) of the estimated mean life span was calculated using the following equation:

$$\text{SE}=\sqrt{\frac{1}{N\left(N\;-\;1\right)}\underset{j}{\sum }{\left(\frac{x_{j}\;+\;x_{j\;+\;1}}{2}\;-\;\text{MLS}\right)}^{2\;}d_{j}}$$

Maximum life span was calculated as the mean life span of the longest-living worms (15%) in each group.

### RNA extraction and RNA sequencing.

Three-day-old animals were cultured on mNGM plates covered with E. coli OP50 or *C. acnes* strains at 25°C for 6 h. Approximately 200 worms per group were collected, washed at least three times with M9 buffer containing 0.2% gelatin, and soaked in RNAlater solution (Qiagen, Hilden, Germany). These samples were stored at −80°C until RNA extraction. Thawed nematode suspensions were ground using a microtube pestle (Scientific Specialties, Inc., Lodi, CA, USA) before being homogenized in TRIzol (Thermo Fisher Scientific, Waltham, MA, USA). Total RNA was isolated using the RNeasy minikit (Qiagen). RNA sequencing was performed using DNAFORM (Yokohama, Japan) as follows: mRNA was purified using the MagnoSphere ultrapure mRNA purification kit (Clontech, Mountain View, CA, USA). Libraries prepared using the SMARTer stranded RNA-Seq kit (Clontech) were run on a HiSeq instrument as 150-bp paired-end reads (Illumina, Inc., San Diego, CA, USA). The quality of RNA-Seq results was assessed using FastQC (version 0.11.7). The raw reads were trimmed and quality-filtered using TrimGalore! (version 0.4.4), Trimmomatic (version 0.36) (46), and cutadapt (version 1.16). Clean reads were aligned against the N2 C. elegans reference genome ce11 (WBcel235.91) using STAR (version 2.6.1a) (47). After the read count of gene features was performed using the featureCounts tool (version 1.6.1) (48), quantitative differential expression analysis between conditions was performed using DESeq (version 1.30.0) (49) to compare control-fed and C. acnes-fed groups. Genes with a |log_2_FC| of ≥2 and *P* value of <0.05 (Tables S1 and S2) were subjected to enrichment analysis based on their Gene Ontology (GO) category for biological process (BP), molecular function (MF), and cellular component (CC) using clusterProfiler (version 3.6.0) (Tables S3 and S4).

### Reverse transcription and real-time PCR.

Synthesis of cDNA was performed using the QuantiTect reverse transcription kit (Qiagen). Real-time PCR (quantitative PCR) was performed in a StepOnePlus real-time PCR system (Thermo Fisher Scientific) using Power SYBR green PCR master mix (Thermo Fisher Scientific) with the following parameters: initial denaturation at 95°C for 10 min, followed by 40 cycles of 95°C for 15 s and 60°C for 1 min. Samples from three biological replicates were analyzed. Relative mRNA expression was determined using the cycle threshold (ΔΔ*C_T_*) method (50) and normalized to the expression of housekeeping genes *act-1*, *tba-1*, and *cyc-1*. Primers used for real-time PCR are listed in Table S5.

### Determination of C. elegans survival time upon infection with S. aureus.

Synchronized L1 nematodes were transferred onto fresh mNGM plates covered with E. coli OP50 or C. acnes and incubated at 25°C for 2 days until worms in each group reached the young-adult stage. Worms were transferred onto an mNGM plate covered with S. aureus (35 worms per plate, two plates for each treatment) (day zero) and incubated at 25°C throughout the observation period. Worm survival was determined using the Kaplan-Meier method, and differences in survival were tested for significance using the log-rank test.

### Measurement of live-bacterial-cell accumulation.

Numbers of E. coli OP50, C. acnes, and S. aureus cells in the nematodes were determined according to the method of Garsin et al. (51), with some modifications. Synchronized L1 nematodes were transferred onto fresh mNGM plates covered with E. coli OP50 or C. acnes and incubated at 25°C for 2 days until worms in each group reached the young-adult stage. Worms were transferred onto mNGM plates covered with S. aureus and incubated at 25°C. On days zero, 1, and 3 postinfection, animals (3 worms/tube) were treated with 500 μl of 1 mg/ml gentamicin in M9 buffer for 30 min. Animals were washed three times with the M9 buffer and mechanically disrupted using a microtube pestle for approximately 30 s. Each suspension was spread on salt egg yolk agar (Nissui Pharmaceutical, Tokyo, Japan) and incubated for 48 h at 37°C for counting of CFU. To measure E. coli OP50 and C. acnes accumulation, suspensions of animals fed E. coli OP50 were incubated on MacConkey agar under aerobic conditions for 24 h at 37°C and those fed C. acnes were incubated on GAM agar under anaerobic conditions for 24 h at 37°C.

### Disk diffusion assay.

The assay was performed in accordance with a previously described protocol (Kissoyan et al. [52]) with some modifications. E. coli OP50 was grown in TSB overnight at 37°C under aerobic conditions, and C. acnes strains were grown in GAM broth for 3 days at 37°C under anaerobic conditions. For preparation of the mixed culture, each broth culture was further incubated with S. aureus overnight at 37°C under aerobic conditions. The optical density at 600 nm (OD_600_) of the bacterial suspension was adjusted to 1.0 using TSB or GAM broth. Then, the culture was centrifuged at 69 × *g* for 5 min and the culture supernatant was filtered with a 0.45-μm syringe filter unit (Millex-HV) to obtain cell-free supernatant. Sterile 10-mm filter paper (Advantec, Japan) was inoculated with 50 μl of the filtered supernatant and placed on S. aureus-inoculated Mueller-Hinton plates. Sensi-Disc ampicillin 10 μg (Becton Dickinson, USA) and Sensi-Disc chloramphenicol 30 μg (Becton Dickinson, USA) were used as positive controls. The plates were incubated overnight at 37°C and visualized.

### Statistical analysis.

The two-tailed *t* test and the log-rank test were used for statistical analyses using Microsoft Excel with the add-in software +Statcel 4 (OMS, Tokorozawa, Japan).

### Data availability.

The raw and processed data files that support the findings of this study are available in Gene Expression Omnibus (GEO) (GSE176406).
